# Supplementary material for: Are carbon emissions trading and green financial instruments synergistic? -Comprehensive quantitative research based on content analysis
Source: PLoS One. 2024 Mar 7;19(3):e0298601. doi: 10.1371/journal.pone.0298601 (PMC10919601; doi:10.1371/journal.pone.0298601)
Supplement: S1 Data — (ZIP) [file pone.0298601.s001.zip › Data/地级市Z_F工作报告.zip!/┴╔─■/╕o╦│╩╨/└┤╫╘╣1⁄2╓┌║┼í╛┬φ┐╦╩2╛▌═°í┐_2009.docx]

各位代表：

　　现在，我代表市政府向大会报告工作，请予审议，并请各位政协委员提出意见。

　　一、2008年政府工作回顾

　　2008年，在市委的正确领导下，在市人大、市政协的监督和支持下，市政府认真贯彻落实科学发展观，带领全市人民抢抓机遇，埋头苦干，战胜各种困难，圆满完成了市十四届人大一次会议确定的各项任务，实现了本届政府工作的良好开局。

　　经济持续快速增长。预计实现地区生产总值662亿元，比上年增长16%；地方财政一般预算收入45.2亿元，增长22.9%；全社会固定资产投资380亿元，增长35%；社会消费品零售总额286亿元，增长21.5%；实际利用外资1.29亿美元，增长88%；城市居民人均可支配收入12430元，增长15%；农民人均纯收入5558元，增长14.6%；万元生产总值能耗降低4.8%。

　　沈抚同城化全面推进。编制完成了沈抚连接带总体发展概念规划、沈抚连接带核心区控详规划，确定了高新区、经济开发区浑河南区和南环路两侧的产业规划，明确了围绕浑河城市段打造“沈抚黄金带，水岸新城区”的发展定位。沈抚同城化标志性项目沈抚城际轨道交通建设全面开工，打通了连接沈抚的高上线，开通了3条公交线路，启动了核心区的水系工程，完成投资20亿元的基础设施建设。企业自主冠名“沈阳”或“抚顺”成为现实。全年招商引资签约合同项目18个，总投资354亿元，是上年的2.4倍。高新区工作机制进一步理顺，基础设施建设步伐加快，投入2.2亿元完成土石方278万立方米，为招商引资、项目落地创造了条件。

　　对外开放实现历史性突破。加大招商引资力度，全年六次赴香港、长三角和珠三角等地招商引资，累计签约项目120项，其中超10亿元的42项，总投资人民币1334亿元，外资12.1亿美元，分别增长4.5倍和5倍。其中，签订正式合同70项，总投资人民币621.5亿元，外资4.9亿美元，分别增长14倍和2倍。外贸出口完成8.6亿美元，增长30%。加大向上争取资金力度，全年累计争取资金44亿元，增长30%。特别是去年第四季度针对国家新增扩大内需1000亿元投资，全市积极策划包装一批项目，争取资金1.66亿元，在全省位居第二。加大向上争取政策力度，积极向上反映抚顺老工业基地遗留的历史问题，在争取我市进入第二批国家资源枯竭型城市转型试点、全国重大地质灾害治理试点市、辽宁省建立大伙房水库上游水资源补偿机制等政策支持方面取得重大进展。

　　经济发展后劲明显增强。以大力发展工业支撑全市经济，工业总产值突破1000亿元。以科技创新引领产业升级，建立石油化工、煤炭、冶金、装备制造等重点产业产学研战略联盟，实现高新技术产业增加值92亿元，增长25%。以大力发展非公有制经济调整所有制结构，新增民营企业1048户。以大力发展服务业调整产业结构，总投资108亿元的丰远·热高乐园、东北科技城、浙商国际商贸城、3个星级酒店等项目陆续开工；总投资65.5亿元的抚顺大剧院、抚顺大酒店、32方块、中兴时代广场二期、橡胶一厂等南站地区改造拉开帷幕；总投资140亿元的东北汽车贸易城、旺力商业城、中欧环保新材料产业园、明达意航商业一条街等项目稳步推进；皇家极地海洋公园室内大型沙滩冲浪馆等龙头旅游项目顺利实施。以大项目建设积蓄发展后劲，开工建设超亿元项目82个，完成投资280亿元，特别是千万吨炼油、百万吨乙烯等大项目建设进展顺利。

　　新农村建设扎实推进。县域经济快速发展，预计三县生产总值、财政一般预算收入增幅分别高于全市平均水平13个和13.7个百分点。粮食产量达到60万吨，创历史最好水平。现代农业建设步伐加快，启动设施农业、畜禽业、林业倍增计划，建设了一批设施农业小区。农村基础设施进一步完善，全市农村宽带覆盖率达到95%，黑色路面实现村村通，有线电视实现屯屯通。除险加固中小型病险水库10座，整治中小河道130公里。开工建设城镇污水处理厂5座，建成村镇卫生填埋垃圾场9座。新建沼气池5070座、秸秆气化站10座。林权配套改革和“国家现代林业示范市”建设取得重大进展，植树造林2.3万公顷，封山育林2万公顷，新开发林地经济2.5万公顷。进一步加大扶贫力度，争取省扶贫资金和动员市直部门帮扶投入近5000万元，建设扶贫项目82个，提高了农民致富能力。

　　城市建设步伐加快。抚顺南站实现整体搬迁，抚顺新站开工建设，长期困扰城市交通的16个平交道口将成为历史。南环铁路建设获铁道部批准，沈吉高速公路南杂木至草市段建设进展顺利，高山路、南环公路全线打通，抚清线和抚金线竣工通车，城市南部、北部和东部出入口瓶颈问题得到解决。改造城市主次干道19条、居住小区路45条、“摸黑路”40条。完成儿童公园、搭连桥和新屯桥改造工程，市长途汽车客运中心站投入使用。加强了市容环境综合整治和重点街路、浑河两岸的美化亮化。强化生态建设，整治浑河城市段及支流河16.6公里，城区春季植树110万株，增长2.9倍；新增绿地170公顷，增长1.2倍。节能减排和环境保护成效明显，化学需氧量、二氧化硫减排超额完成年度目标，大伙房水库水质保持二类水体标准。土地资源的节约集约利用全面加强，实现土地出让收入27.6亿元，增长83.7%。

　　和谐抚顺建设迈出新步伐。坚持尽心竭力为群众办实事，让改革发展的成果最大限度地惠及百姓。不断改善群众住房条件，拆迁棚户区37.2万平方米，建设安置用房70万平方米，城市集中连片棚户区改造基本完成。启动农民迁移式搬迁，全市新建住宅44万平方米，1.6万农民喜迁新居。加强社会保障体系建设，社会保险扩面征缴专项行动成效显著，征缴各项保险费26.4亿元,增长27.3%；其中养老保险统筹基金13亿元，增长37.4%，首次实现收入增幅高于支出增幅，确保了26.7万离退休职工养老金按时足额发放。争取国家和省资金7.5亿元,解决困难企业拖欠职工工资和退休职工医疗保险问题。启动城镇居民医疗保险，覆盖率达50%。建立完善城乡社会救助体系，提高了城市低保标准,为近20万城乡低保居民、5500名农村五保对象发放最低生活保障金2.8亿元。加大就业政策落实力度,为“4050”人员等困难群体发放政策性资金补贴2.6亿元。投资4000多万元，建设城乡两级就业平台，充分就业社区达到60%以上，全年新增实名制就业13.5万人，城镇登记失业率控制在4.1%以下。改造1万户旧有房屋供水设施，解决了2680户城市居民吃水难和6.7万农民饮水安全问题。全力维护社会稳定，投入3.5亿元，解决了1074个信访案件，信访总量下降36.7%。妥善处置了蚁力神破产清退、东华集团非法集资和一大批历史积案。强化以煤矿、尾矿库和化工企业为重点的安全隐患排查治理，各类事故起数和死亡人数分别下降41.8%和20.8%。强化奥运安保，推进社会治安防控体系建设，保持对各类犯罪的高压严打态势，全市命案和刑事案件发案率分别下降20%和16.8%，人民群众的安全感进一步增强。先后派遣12批专业工作队赴四川地震灾区对口支援，全市为灾区捐赠1.1亿元。

　　社会事业全面进步。各级各类教育健康发展，教育教学成果显著，全市高考录取率达80.6%，高于全省平均水平4.1个百分点。职业教育特色专业不断加强，高等职业和中等职业学校毕业生就业率分别达到91%和95%。新建和维修农村中小学校舍41所，彻底消除教育危房。筹集资金797万元，资助贫困学生2万人，使121名“宏志生”圆了大学梦。举办广场文化演出2100场，煤精雕刻、满族剪纸被列入国家级非物质文化遗产保护名录。公共卫生服务体系和医疗服务体系建设进一步加强，新型农村合作医疗覆盖率达100％，农民参合率达97%；新建和改建乡镇卫生院20所、村卫生室194个。积极应对“问题奶粉事件”，清查经营业户2.8万户次，免费排查患儿1.67万人次，危重病患儿为零。抚顺籍运动员在北京奥运会、残奥会上取得2金2银的好成绩，承办第十五届全国冬泳锦标赛获得圆满成功。人口增长继续保持低生育水平，人口自然增长率为0.38‰。民族宗教、审计、统计、人防、粮食、物价、供销、外事（侨务）、对台、地震、气象、档案、史志、妇女儿童、老年人、残疾人、双拥共建、优抚安置等各项工作都取得了新的进展。

　　政府自身建设成效显著。自觉接受人大及其常委会的法律监督、工作监督和政协的民主监督，办理人大代表建议324件、政协委员提案568件，办结率达100%，满意和基本满意率达99.7%。结合环境年建设，制定出台了对重大项目、重点企业实施保护和鼓励企业上市融资发展等一系列政策规定，并对矿山采选业、建筑业等重点领域，集中开展了经济秩序综合整治，查补税费3.6亿元，市场环境明显改善。坚持市长办公例会制度，推进行政决策的科学化、民主化。大幅减少行政审批项目，政府工作效率明显提高。积极偿还历史债务，开展大规模的增收节支活动，保证了建设需要和机关正常运转。切实转变工作作风，争分夺秒、夙兴夜寐，求发展、谋振兴的氛围已经形成。

　　各位代表，过去的一年，是全市经济快速增长，社会全面协调发展的一年；是招商引资取得显著成果，对外开放取得重大突破的一年；是城市建设步伐加快，发展环境进一步改善的一年；是群众生活不断提高，和谐抚顺建设取得重大进展的一年。这些成绩的取得，得益于市委的正确领导和市人大、市政协的大力支持，得益于中省直单位和市属国有、民营企业的巨大贡献，得益于各县区、各部门的辛勤努力，得益于全市广大干部的只争朝夕、埋头苦干，得益于全市各族人民的努力拼搏、团结奋斗。在此，我谨代表市政府，向为抚顺发展付出艰辛、作出贡献的各位代表和委员，向奋战在全市各条战线的广大干部群众，向各民主党派、工商联、无党派人士、人民团体，向中省直单位、驻抚部队、武警官兵和老领导、老同志，向所有关心支持抚顺发展的海内外朋友表示衷心的感谢和崇高的敬意！

　　在肯定成绩的同时，我们也清醒地认识到，我市经济社会发展还存在许多矛盾和问题。一是经济结构性矛盾仍然突出。产业结构、产品结构不尽合理，服务业比重小，产业链条短，产品精深加工水平低；开放程度不高，战略性和牵动性的大项目少；发展方式粗放，乱占私采滥用资源的现象仍然存在。二是历史遗留问题突出。采煤引发的地质灾害严重，政府债务沉重，养老保险金缺口巨大，国企改革滞后，城市建设欠账较多。三是涉及人民群众切身利益的问题还比较突出。冬季供暖、社会保障、医疗服务、土地征用、房屋拆迁、安全生产等方面，还存在群众不满意的地方。四是政府职能有待进一步转变。一些部门和机关干部思想不解放，加快发展的紧迫感和责任感不强，推诿扯皮，吃拿卡要，办事效率低下的现象仍然存在。对于这些矛盾和问题，我们必须以对抚顺发展高度负责的精神，采取更加有力的措施，认真加以解决，决不辜负全市人民的期望。

　　二、2009年政府工作目标与任务

　　当前，受国际金融危机日趋严重的影响，我市面临的形势将十分严峻。去年四季度以来，我市实体经济特别是工业经济受到严重冲击，重点工业产品价格降幅超过40%，导致全市税收锐减，主要经济指标持续回落。这场危机尚未见底，对经济的影响将进一步加剧。做好今年的工作，必须对复杂多变的形势做出正确的判断，既要看到面临的严重困难和严峻挑战，又要看到面临的发展机遇和有利条件；必须积极应对，逆势而上，化危机为机遇，变挑战为动力；必须继续保持昂扬向上的精神状态，以全新的思维、超常的勇气、加倍的努力，扎实推进各项工作，全力保增长、调结构、促和谐，努力实现经济社会大发展、快发展，向全市人民交出一份满意的答卷。

　　今年全市经济社会发展的总体要求是：认真贯彻党的十七大和十七届三中全会精神，深入贯彻落实科学发展观，把促进经济平稳较快增长作为首要任务，全面推进沈抚同城化建设，全力打造“两区两带”，大力推进改革开放和科技创新，着力转变经济发展方式和提高经济增长质量，切实加强节能减排和生态环境保护，更加重视民生改善和社会建设，继续保持经济社会又好又快发展的势头，为3－5年再造一个抚顺奠定坚实基础。

　　今年全市经济发展的主要预期目标是：地区生产总值增长12%，地方财政一般预算收入同口径增长17.6%，全社会固定资产投资增长35％，实际利用外资增长20%，城市居民人均可支配收入增长12%，农民人均纯收入增长10%，万元生产总值能耗降低4.8%。

　　为实现上述目标，重点做好以下八个方面工作：

　　（一）加快“两区两带”建设，大力推进沈抚同城化

　　建设“两区两带”是推进沈抚同城化发展的重要战略，是实现3－5年再造一个抚顺的重要支撑。“两区两带”即西部沈抚同城新区、东部国家精细化工产业区、南环循环经济产业带、浑河生态商住景观带。西部沈抚同城新区，规划面积258.8平方公里，由经济开发区、拉古和石文地区、方晓地区、工农和塔峪地区构成；东部国家精细化工产业区，规划面积50平方公里，由高新区和兰山工业园区构成；南环循环经济产业带，规划面积42平方公里，由南环公路和南环铁路两侧沿线构成；浑河生态商住景观带，规划面积33.3平方公里，由浑河两岸、轻轨两侧沿线构成。加快“两区两带”产业发展和人居环境改善，重点打造世界级炼化基地、世界级油母页岩深加工基地、国家级精细化工产业基地和国家级新材料产业基地，重点打造浑河两岸一道靓丽的风景线。

　　加快推进沈抚同城新区建设。加强核心区和产业区基础设施建设，投入22亿元完成“四横三纵”路网建设，打造浑河、李石河景观带和核心区南湖、北湖水系，形成“两景两湖”景观。加强生态区基础设施建设，投入10亿元完成重点项目配套工程。重点推进总投资超200亿元的丰远· 热高乐园等6个商贸旅游项目，稳步推进总投资40亿元的欧洲工业园等18个工业项目，积极推进总投资55亿元的锦绣澜湾等9个房地产项目。确保全年固定资产投资200亿元，新开工超亿元项目20个。

　　加快推进国家精细化工产业区建设。立足园区开发市场化、投资主体多元化，重点加快起步区中3.22平方公里的基础设施建设，尽快实现“三通一平”，达到项目摆放条件。依托千万吨炼油、百万吨乙烯项目，拉长产业链，形成产业集群，力争引进2家世界500强企业落户。

　　加快推进南环循环经济产业带建设。以南环公路全线贯通和南环铁路建设为契机，加快两侧特别是9平方公里起步区的基础设施建设，积极推进投资60亿元的120万千瓦煤矸石热电厂、10亿元的页岩油化工综合利用项目主体工程和15亿元的油母页岩小颗粒炼油装置项目建设，争取国家批复建立油母页岩研发中心。积极推进投资60亿元的抚顺发电厂、50亿元的新钢铁搬迁改造、30亿元的60万千瓦抚顺铝业自备电厂和15亿元的万吨海绵钛等项目开工建设，努力引进一批大项目和产业集群。

　　加快推进浑河生态商住景观带建设。以“沈抚黄金带、水岸新城区”为主题，按照总体规划、分步实施的原则，全力打造浑河生态商住景观带。在浑河两岸和沈抚城际轨道沿线，重点规划建设南站商业区北区、325库、方晓新城、月牙岛、绥化路北、将军沟、高山路两侧等10余项大型综合开发项目。加快推进浑河中部段滨河公园、浑河北岸滨河路建设，实现浑河中部、西部局部河段游船通航。完成月牙岛及其两岸地区的规划、防洪和土地整理等前期准备工作，达到项目摆放条件。

　　（二）全方位对外开放，实现招商引资新突破

　　强化招商引资。做好今年的工作，核心在于扩大投资，重点在于招商引资，关键在于项目建设。要集中力量，引进重大项目和产业集群。全年引进超千万美元的外资项目10个，引进超亿元的内资项目50个，引进超10亿元的内资项目10个，各县区和开发区都要承接一个产业集群项目。强化项目落地工作机制，建立重点项目“绿色通道”，使签约项目早开工，开工项目早投产。

　　积极争取资金。紧紧抓住国家扩大内需的重大机遇，做好项目的策划包装和评估论证，积极向国家申报产业结构调整、基础设施建设、城市改造和民生工程项目，争取国家和省更多的资金支持，确保争取国家和省资金的额度在全省各市平均水平以上。今后政府新建项目所需资金，原则上以向上争取和市场化运作为主，以财政资金或贷款配套为辅，实现负债额度与偿债能力的总体平衡。

　　积极争取政策。密切与国家和省有关部门的沟通联系，全方位争取国家和省的政策支持，确保我市列入第二批国家资源枯竭型城市转型试点，争取列入全国重大地质灾害治理试点市，并在建立大伙房水库上游水资源补偿机制、扶持县域经济发展、加快工业园区基础设施建设等方面，争取更多的政策支持。

　　（三）强力推进国企改革，发展壮大民营经济

　　做大做强骨干企业。坚持扶优扶强，重点支持10户销售收入超10亿元的企业，抓好一批重大工业项目建设，年内规模以上工业企业突破1000户。充分发挥科研院所作用，大力推进科技创新，提高企业核心竞争力，实现高新技术产业增加值110亿元，增长20%。建立银企对接长效机制，促进互惠共赢。落实鼓励企业上市融资有关政策，重点做好4户企业上市准备和10户企业上市培育工作。

　　加大国企改革力度。按照“属地为主、因企制宜、规范操作、不留后患”的原则，成熟一个改制一个。以政策性破产和依法破产为主，积极争取国家和省政策支持，全力推进8户国有企业政策性破产，积极推进122户国有企业改制，逐步解决97户已改制企业的遗留问题。

　　促进民营经济快速发展。强化政策支持，优化服务环境，大力发展非公有制经济。鼓励和支持民营企业以股份制形式进行重组整合，以多种形式参与国企改革。加强中小企业融资担保体系建设，每个县区至少成立2家担保公司，力争全市达到20家，切实解决中小企业融资难问题。

　　（四）大力发展服务业，积极培育新的经济增长点

　　加快发展商贸流通业。优化商业布局，打造新抚区、经济开发区、顺城区和望花区4个商务中心区，启动南站商业区改造，重点推进投资10亿元以上的10个商贸项目建设，积极推进华山、裕民物流园区建设，全市社会消费品零售总额增长17%。

　　加快发展旅游业。进一步整合旅游资源，围绕清永陵、赫图阿拉城等清前史迹和满族民俗资源，打造文化旅游区；围绕红河峡谷漂流、皇家极地海洋公园、萨尔浒风景区、丰远·热高乐园，打造休闲旅游区；围绕三块石、猴石、和睦、岗山、老秃顶、浑河源、莲花山和金山石佛，打造生态旅游区；围绕雷锋纪念馆、平顶山惨案遗址纪念馆、抚顺战犯管理所，打造红色旅游区；围绕抚矿集团、石化公司等工业企业和工业遗产，打造工业旅游区。积极推进清原县、新宾县、抚顺县星级酒店等旅游设施建设，全年旅游总收入增长26%。

　　加快发展房地产业。坚持住宅地产、商业地产和工业地产同步发展，全面活跃房地产交易市场。立足山水资源优势，打造浑河生态商住景观带。按照政府主导、规划先行、市场运作、成片开发的原则，制定切实可行的政策，全面启动城区危旧楼改造工程。加快县城房地产业发展，确保房地产销售额增幅不低于15%。

　　加快发展金融业。抓住国家实施适度宽松货币政策的机遇，鼓励各类金融机构扩大信贷规模，认真解决存贷差过大问题，实现全市新增贷款规模高于新增存款规模。市商业银行要在增资扩股、完善公司治理结构的基础上尽快做大做强。每个县区至少成立1家小额贷款公司，力争全市小额贷款公司达到10家，力争在抚顺县和经济开发区开办2家村镇银行。

　　（五）发展壮大县域经济，扎实推进社会主义新农村建设

　　大力实施强县战略。围绕县域经济三年翻番目标，搞好县域经济发展规划，每个县都要确立1－2个特色主导产业，引进一个有影响力的产业集群，引进一批具有牵动力的重大项目。加快推进抚顺县拉古和兰山工业园、清原县健康食品加工园和孤山子万亩工业园、新宾县南杂木工业园和永陵特艺木业园建设。积极争取并用好、用足、用活相关政策，促进县域经济快速发展。

　　加快发展现代农业。按照“一县一业”、“一县一品”的要求，重点推进百亿产值的良种奶牛繁育产业集群项目建设，建设241个设施农业小区、178个畜牧业养殖小区。深化林权配套改革，全面推进现代林业建设，新开发林地经济2.7万公顷。大力推进农业标准化，积极发展农民专业合作组织。按照依法自愿有偿的原则，积极推进土地使用权、林地经营权和林木所有权的有序流转，实现规模化、集约化经营。

　　加强农业基础设施建设。重点抓好10座中小型病险水库除险加固和364项农田水利设施建设等工程，加快推进3座农村小水电站、10处生物气化工程和4500个“一池三改”等能源工程建设，全面实施2667公顷中低产田改造和1万公顷小流域治理等农田综合整治工程，提高农业防灾减灾能力。

　　（六）加强基础设施和生态环境建设，为又好又快发展奠定坚实基础

　　坚持科学规划城市。以新一轮城市总体规划修编为契机，高起点编制4个城区的分区规划和沈抚连接带、浑河两岸、沈抚城际轨道沿线、南环产业带、采煤引发地质灾害影响区综合利用等专项规划，提高控详规划覆盖率。加强对县城和村镇规划的指导。统一编制水、电、气、通信等管网规划，统一实施管线埋设，避免重复建设。

　　加快城市基础设施建设。加快沈抚城际轨道交通建设，确保5月1日竣工通车。积极推进南环铁路、永陵至桓仁、南杂木至通化高速公路建设的启动，加快推进南杂木至草市段沈吉高速公路建设。完成南环公路建设，启动东环大道建设，打通南北通道，年内形成城市环城公路。加快垃圾处理场建设，改造新建“三供”管网60公里、输水暗渠10公里，改造城市道路45条、“摸黑路”40条。完成城东二期基础设施建设，基本完成以居民脱险为重点的第三期采煤引发地质灾害治理任务，全面完成城市集中连片棚户区改造收尾工程。继续实施环城林带造林补植工程，新造林补植600公顷，完成城区植树100万株。

　　强化城市管理。推进城市管理重心下移和事权下放，充分发挥城区政府管理城市的职能作用，建立区级城市管理和行政执法责任制，完善考评体系。综合整治城市市容环境，严肃查处违法建设和非法营运行为。城市主次干道人工清扫保洁率、生活垃圾清运率达到国家标准。大力推进土地节约集约利用，搞好基本农田布局调整，保证建设项目用地。规范土地市场，严格执行用地招拍挂制度，实现土地出让收入35亿元，增长26.8%。

　　积极推进污染整治。坚持不懈地抓好节能减排，力争提前实现省政府下达的减排目标。建成大伙房水库上游5座污水处理厂，关停危害重点水源地的工矿企业，确保饮用水源地水质安全。继续实施望花等重点区域大气综合整治，加快推进东洲、望花2座再生水厂和“一镇一热源”建设。充分利用抚顺热电公司的热源，实行集中供热，从8月1日起在城区全面禁用1吨以下燃煤锅炉。投入1000万元，全力开展大气污染整治，全面提高城市空气质量。

　　（七）着力推进重点民生工程，为全市人民创造更多福祉

　　努力拓宽就业渠道。统筹城乡就业，强化失业预警制度建设，鼓励自主创业，降低贷款门槛，实名制扶持创业带头人400人。对零就业家庭、困难家庭大学毕业生和大龄就业困难人员实行“兜底”安置。加强就业培训和组织劳务输出，普惠制培训2万人，实名制劳务输出2.1万人。充分就业社区达到75%以上，完成实名制就业9万人以上，城镇登记失业率控制在4.5%以下。

　　强化保障体系建设。加大以养老保险为重点的社会保险扩面征缴，实现应收尽收、应保尽保。调增企业离退休人员养老金，确保养老金按时足额发放。全面推进城镇居民基本医疗保障体系建设，完成城镇居民医疗保险扩面任务。继续解决好困难企业退休人员医疗保险问题。建立新型农村养老保险制度、农村低保边缘户救助制度和失地农民社会保障平台，适时提高城市低保标准。实施“蓓蕾生命”慈善救助工程，使全市217名困难家庭患病儿童得到救治。建成流浪少年儿童救助保护中心。加快农村贫困户危房改造和饮水工程建设，抓好新一轮整村推进扶贫30个，移民扶贫260户。完成水库移民6万人的直补和项目扶持。

大力发展社会事业。坚持义务教育体现公平均衡、职业教育突出优势、各类学校办出特色的原则，调整教育布局，整合教育资源，推进教育协调发展。加强公共卫生和医疗服务体系建设，完善社区卫生服务机构功能，改善县医院和乡镇卫生院条件，提高诊治水平。继续稳定低生育水平，不断提高出生人口素质。加强文化设施建设，异地改建抚顺大剧院、青少年宫等文化场所，建设10座乡镇文化站、1600户文化信息资源共享服务点。推进数字电视平移2万户，城乡宽带覆盖率达到98%。加强体育场馆建设，新建健身场所120处，承办好全国青少年乒乓球比赛。加强老干部工作，做好民族宗教工作。维护妇女、儿童、老年人和残疾人的合法权益。加强精神文明建设，大力弘扬雷锋精神，争创全国双拥模范城和省级文明城市。

切实维护社会稳定。以争创全国综治工作优秀市为目标，强化社会治安综合治理，健全治安防控体系，严厉打击各种犯罪，进一步增强群众的安全感。加强群众来信来访工作，健全群众利益诉求表达和矛盾排查调处机制，依照法律和政策及时解决群众反映的问题，扎实推进和谐社区(村)建设，确保社会和谐稳定。加强安全生产，保障食品和药品安全，坚决遏制重特大事故发生。

　　(八)切实加强政府自身建设，打造为民务实高效廉洁政府

　　政府要为民。坚持以人为本、执政为民，把实现好、维护好、发展好人民群众的根本利益作为政府工作的出发点和落脚点，努力做到为人民服务、受人民监督、对人民负责、让人民满意。加强民主法制建设，全面推行政务公开，切实保证人民群众的知情权、参与权和监督权。全面推进依法行政，切实提高政府的执行力和公信力，优化经济发展软环境。自觉接受市人大及其常委会的法律监督和工作监督，自觉接受市政协的民主监督和社会的公众监督，认真办理人大代表议案、建议和政协委员提案,不断提高办结率和满意率。为充分发挥人大代表和政协委员的日常监督作用，政府将随时受理代表和委员的建议和意见。

　　政府要务实。坚持深入实际，调查研究，做到问政于民、问需于民、问计于民。树立过紧日子思想，坚持厉行节约，把有限的资金用在促进发展和改善民生上。坚持说实话、办实事、求实效，提倡开短会、发短文、讲短话，多做打基础、利长远的工作，着力解决影响改革发展稳定的突出问题。认真负责，敢于碰硬，遇到困难不退缩，碰到矛盾不回避。切实把心思放在发展上，把气力用在落实上，把项目一个一个抓实，把事情一件一件办好，努力做出经得起实践、人民、历史检验的实绩。

　　政府要高效。整合行政机关资源，深化行政审批制度改革，提高行政效能，坚决杜绝一件事情让群众跑来跑去、领导批来批去、部门转来转去的推诿扯皮现象，每个政府工作人员必须做到决不让工作在我这里被延误，决不让客商在我这里遭冷落，决不让政府形象在我这里受影响。进一步完善政府绩效考评体系，严格执行首问负责制、限时办结制和责任追究制，提高办事效率，提供一流服务，创造一流业绩。

　　政府要廉洁。坚持从严治政，强化对国有资产管理、政府采购、土地出让、建设工程招投标和代建制、项目规划审批等重点领域的制度建设，确保权力在阳光下运行。严肃查处各类违法违纪案件，决不让腐败分子有藏身之地，决不让腐败问题影响抚顺的整体形象，决不让腐败问题影响抚顺的振兴大业。

　　各位代表，加快抚顺的发展振兴，是时代赋予我们的光荣使命和神圣职责，是全市人民对我们的殷切期望。让我们在省委、省政府和市委的领导下，坚持以科学发展观为指导，带领全市人民团结拼搏，埋头苦干，迎难而上，为3－5年再造一个抚顺，开创抚顺美好的明天而努力奋斗！
